# 380. A molecular epidemiological exploration of reduced vancomycin susceptibility in *Clostridioides difficile*

**DOI:** 10.1093/ofid/ofac492.458

**Published:** 2022-12-15

**Authors:** Taryn A Eubank, Chetna Dureja, Julian G Hurdle, Kevin W Garey, Anne J Gonzales-Luna

**Affiliations:** University of Houston College of Pharmacy, Houston, Texas; Texas A&M Health Science Center Institute of Biosciences and Technology, Houston, Texas; Texas A&M Health Science Center Institute of Biosciences and Technology, Houston, Texas; University of Houston College of Pharmacy, Houston, Texas; University of Houston, Houston, Texas

## Abstract

**Background:**

Use of vancomycin to treat *Clostridioides difficile* infection (CDI) has increased following recent IDSA/SHEA treatment guideline updates, applying a selection pressure for resistance development. We previously demonstrated acquired mutations in VanSR two-component system led to constitutive *vanG* expression and improved *in vitro C. difficile* survival in physiologic vancomycin concentrations. We aim to describe the molecular epidemiology of reduced vancomycin susceptibility in clinical isolates during a period of high vancomycin use.

**Methods:**

A cohort study was performed including adult patients hospitalized with CDI in two health systems (14 hospitals) in the Houston Area between 2017-2021. (Stool transport) *C. difficile* were ribotyped by fluorescent PCR and susceptibility tested by agar dilution in accordance with CLSI standards. Reduced vancomycin susceptibility was defined by minimum inhibitory concentrations (MICs) >2 mg/L. Sanger sequencing was conducted on a subgroup of isolates to identify VanSR mutations. Analysis using Chi square was performed using IBM SPSS Statistics (v 28.0.1.0).

**Results:**

A total of 36% (165/465) of isolates exhibited reduced vancomycin susceptibility (MIC_50_ = 2 mg/L, MIC_90_ = 4 mg/L, range 0.5-16 mg/L), of which 348 were ribotyped. A significantly higher proportion of ribotype (RT) 027 isolates demonstrated reduced vancomycin susceptibility (83%) compared to other common ribotypes (30%); p< 0.001). No differences based on collection year (p=0.3) or healthcare system (p=0.08) were observed. Overall, 11% (7/56) of isolates exhibiting mutations in VanS (n=1), VanR (n=5), or both (n=1). VanSR mutations were present in 47% (7/15) of those with MICs >2mg/L vs 0% (0/41) of those with MICs ≤2 mg/L (p< 0.001).

**Conclusion:**

A high proportion of clinical *C. difficile* isolates exhibited elevated MICs to vancomycin, which was most common in RT027 isolates. Mutations in the *vanG* regulator, VanSR, correlated with elevated MICs in a subgroup of isolates. Future research is needed to expand upon molecular mechanisms and clinical implications of reduced vancomycin susceptibility.

**Disclosures:**

**Kevin W. Garey, PharmD, MS**, Acurx Pharmaceuticals: Grant/Research Support|Paratek Pharmaceuticals: Grant/Research Support|Seres Therapeutics: Grant/Research Support|Summit Pharmaceuticals: Grant/Research Support.

